# DIGNITY: Case Study of Person-Centered Risk Management in Dementia Care

**DOI:** 10.1093/geroni/igaf122.2070

**Published:** 2025-12-31

**Authors:** Caroline Madrigal, Joanne Roman Jones, Mary Lou Ciolfi, Karolus Wangi, Susan Ryan, Nicole Osevala, Liza Behrens

**Affiliations:** VA Boston Healthcare System, Boston, Massachusetts, United States; University of Massachusetts Boston, Boston, Massachusetts, United States; UMaine Center on Aging, Orono, Maine, United States; The Pennsylvania State University, University Park, Pennsylvania, United States; Center for Innovation, Linthicum, Maryland, United States; Penn State Health, Hummelstown, Pennsylvania, United States; Pennsylvania State University, UNIVERSITY PARK, Pennsylvania, United States

## Abstract

While developing the DIGNITY Pilot program, nursing home regulators, leaders, and staff all expressed difficulty with balancing resident autonomy with safety for intimacy preferences of residents living with dementia. Intimacy preferences are challenging to honor in nursing homes due to staff comfort levels with residents’ ability to make autonomous choices, societal stigma surrounding sexuality in older adults, and restrictive policies. The DIGNITY program empowers nursing home staff to collaborate with residents, their families, and other interdisciplinary team members to identify and mitigate risks to the health and safety of residents. This presentation will provide a case exemplar to demonstrate the use of the DIGNITY program for a resident living with dementia who is perceived by staff to be making a risky choice to co-sleep with another resident living with dementia. The scenario will employ the six components of the DIGNITY program, highlighting areas of concern for all involved, namely the resident’s capacity to make informed choices. We will explore the resident’s preferences, values, and capacity for decision-making, utilizing DIGNITY assessment tools and care planning process; and discuss the importance of assessing a resident’s capacity to make informed choices, focusing on their ability to understand, appreciate, and reason about their preferences, distinct from the legal determination of competency. We will discuss potential practice and legal implications for nursing home staff to adopt and implement the DIGNITY process.

